# Fiber Protein Produced in *Escherichia coli* as a Subunit Vaccine Candidate Against Egg-Drop Syndrome 76

**DOI:** 10.3389/fvets.2022.819217

**Published:** 2022-02-25

**Authors:** Linguo Wang, Pantao Zhang, Baicheng Huang, Mengyue Wang, Hui Tian, Peng Liu, Wujie Liu, Kegong Tian

**Affiliations:** ^1^College of Veterinary Medicine, Henan Agricultural University, Zhengzhou, China; ^2^National Research Center for Veterinary Medicine, Luoyang, China

**Keywords:** fiber protein, immunogenicity, subunit vaccine candidate, *Escherichia coli*, duck atadenovirus A

## Abstract

The egg-drop syndrome ‘76 (EDS ‘76) caused by duck atadenovirus A (DAdV-1) infection in laying hens leads to the decrease in egg production, causing heavy economic losses in the poultry industry; thus, vaccines with high safety and immunogenicity are needed. In this study, the DAdV-1 fiber protein expressed in *Escherichia coli* with codon optimization showed the hemagglutination (HA) titer of 13 log2 after purification (0.6 mg/mL). Compared with inactivated EDS ‘76 vaccine, the specific pathogen-free chickens immunized with 0.4 mL fiber protein (HA titer of 11 log2) induced an equal level of HA inhibition (HI) titer and neutralizing antibodies. Meanwhile, after immunization with fiber protein, the lowest HI titer that could provide the effect to reduce egg production rate in laying hens after the challenge was 7 log2. Moreover, fiber protein with an HA titer of 7 log2 could induce an HI titer no <7 log2 in laying hens, which was equal to or higher than the lowest HI titer (7 log2) that could reduce egg production against DAdV-1 infection significantly, indicating that it is economically feasible for vaccine development. Importantly, the HI antibodies maintained at a high level up to 180 days postimmunization contribute to the clinical application of the vaccine candidate. Overall, the fiber protein produced in *E. coli* is an effective subunit vaccine candidate in EDS ‘76 control for its high immunogenicity and protection in chickens.

## Introduction

Duck atadenovirus A, also known as duck adenovirus A (DAdV-1), is responsible for the egg-drop syndrome ‘76 (EDS ‘76) in laying hens. DAdV-1 is a member of the genus *Atadenovirus* within the family Adenoviridae, with an ~30- to 35-kb linear double-stranded DNA genome (1). EDS ‘76 was first reported in 1976 (2); only one serotype was found. DAdV-1 mainly infects waterfowl like its natural host duck and geese (3). In laying hens, EDS ‘76 causes a significant drop of egg production and the low quality of the eggs (4, 5). Vaccination is effective in the prevention and control of EDS ‘76; birds immunized with the inactivated EDS vaccine were protected after challenge and laid normally (6). The capsid protein of DAdV-1 contains important adenovirus-neutralizing epitopes (7). Therefore, for higher safety and immunogenicity in the vaccination, the DAdV-1 capsid protein was being used in the development of the subunit vaccine (7, 8).

Fiber protein, a component of DAdV-1 capsid, has been proven to be the most effectively antigen to induce virus-neutralizing antibodies (9, 10), which has three domains: N-terminal tail, a long shaft, and C terminal globular head (knob) domain (11–19). The knob domain contains most of the neutralizing epitopes, involved in the attachment of DAdV-1 to host cells (20); thus, it is a suitable target for subunit vaccine development. The knob domain including part of the shaft region expressed in *Escherichia coli* induced the production of HA inhibition (HI) and neutralizing antibodies in immunized chickens (21, 22). After fusing with chicken cartilage matrix protein (CMP), the shaft sequence lacked knob protein (CMP-knob) was effective to induce HI antibodies compared with the inactivated EDS vaccine, providing the protection for laying hens against DAdV-1 infection (8). The difficulty in purification of the recombinant knob protein in inclusion bodies makes it costly for subunit vaccine development (21). The knob domain with part of the shaft region could be soluble expressed in *E. coli*, and stimulated HI and neutralizing antibody responses in immunized specific pathogen-free (SPF) chickens. Moreover, immunization with the fiber protein also induced lymphocyte proliferation response, cytokine secretion, and reduced viral load in chicken (23). However, there were no sufficient data about vaccine efficacy in laying hens.

In this study, the fiber protein of an isolated DAdV-1 strain was expressed in *E. coli* expression system, and then the ability to induce HI and neutralizing antibodies was tested in SPF chickens. Moreover, we evaluated the HI titer of the serum from laying hens immunized with fiber protein, and the HI titer of the serum and egg production of laying hens after challenge.

## Materials and Methods

### Animals

SPF chickens and laying hens (Hy-Line Brown that is DAdV-1 antibody negative and with normal egg production rate) were housed in containment cages in a biosafety level 2 animal facility. All the animal samples were collected according to the protocol approved by the Animal Care and Ethics Committee of National Research Center for Veterinary Medicine (permit 20170625006).

### Virus

DAdV-1 AV127 strain (GenBank accession no. Y09598) was propagated in duck embryo fibroblasts. DAdV-1 HX strain was isolated from the oviduct of a laying hen with the soft-shelled and non-shelled egg production in Henan province and propagated in the allantoic fluid of 10-day-old SPF embryonated duck eggs at 37°C for 120 h.

### Plasmid Construction and Expression of Fiber Protein

The HX strain fiber gene (Supplementary Figure 1) was synthesized with the codon usage bias modification that targeted *E. coli* for a higher expression profile, and then the gene was amplified by polymerase chain reaction (PCR) using the primers of fiber-F, CATG*CCATGG*GCATGAAGCGACTACG, and fiber-R, CCG*CTCGAG*CTACTGTGCTCCAACATA; the restriction sites of *Nco*I and *Xho*I (NEB, USA) are shown in italics. Subsequently, the purified PCR product was digested with *Nco*I and *Xho*I and then cloned into pET28a plasmid. After the confirmation by sequencing, the positive clone was transformed into *E. coli* BL21 (DE3) and then cultured in LB medium with 50 μg/mL of kanamycin, the protein expression was induced with 0.2 mmol/L of isopropyl-β-d-thiogalactoside (IPTG) at 20°C. The cell pellets resuspended in lysis buffer (20 mM Tris, pH 8; 250 mM NaCl) were lysed by sonication and then centrifuged to remove the insoluble pellet. The expression and distribution of fiber protein were analyzed by sodium dodecyl sulfate–polyacrylamide gel electrophoresis (SDS-PAGE) and Western blot. Standard chicken antiserum against DAdV-1 strain AV127 (China Institute of Veterinary Drug Control, Beijing, China) was used as primary antibody in Western blot. After that, the fiber protein was purified according to the instruction of Ni-NTA Purification system (Invitrogen, USA). The working flow of the fiber protein expression is shown in Supplementary Figure 1.

### Hemagglutination Assay

We used HA titer to evaluate the quantity of fiber protein in this study. The fiber protein was serial 2-fold diluted by phosphate-buffered saline (PBS; pH 7.2) and added in wells of the 96-well V-shaped microtiter plates (25 μL/well). Subsequently, each well was added with an equal volume of 1% (vol/vol) chicken erythrocyte suspension and incubated at room temperature (RT) for 30 min. The HA titer was expressed as the reciprocal of the highest virus dilution showing complete agglutination.

### Serum Neutralization Test

Neutralizing antibody titers of the serum from the immunized SPF chickens were detected by serum neutralization test as descripted previously (21). Briefly, after immunization, 0.5-mL serum samples of the chickens collected at different timepoints were inactivated at 56°C for 30 min and incubated with equal volume of 10^5.1^ EID_50_/mL of AV127 at 37°C for 1 h. Subsequently, 0.2 mL serum/virus mixture was inoculated into the allantoic cavity of six SPF embryonated duck eggs (10 days old), which were then incubated at 37°C for 7 days and candled daily. After that, the allantoic fluid of the SPF embryonated duck eggs was collected for HA tests. Results are expressed as percent eggs in which the virus was neutralized.

### Hemagglutination Inhibition Assay

In HI assay, serum samples were serial twofold diluted by PBS (pH 7.2) and added in wells of the 96-well V-shaped microtiter plates. Each dilution was mixed with 4 hemagglutinating units of inactivated DAdV-1 of AV127 strain. After incubation for 15 min at RT, each well was added with an equal volume of 1% (vol/vol) chicken erythrocyte suspension and incubated at RT for 30 min. The highest serum dilutions that showed HI activity were determined as the HI titers.

### Animal Experiment Plan

To test the HI and neutralization activity of the serum from immunized SPF chickens, the fiber protein was quantified to HA titer of 11 log2 by diluting with the PBS. The quantified fiber protein was mixed with oil adjuvant at a ratio of 2:1 and then emulsified to generate water-in-oil emulsions for immunization. Briefly, in the three groups (10 per group, 28 days old), the SPF chickens in group 1 were administered with fiber protein (0.4 mL/chicken) intramuscularly, and the chickens in group 2 were administered with inactivated EDS vaccine, and group 3 was set as the unimmunized group. Serum samples collected at 14, 21, and 28 days postimmunization (dpi) were tested by HI and neutralization assay.

To test the subunit vaccine efficacy, the laying hens (Hy-Line Brown, *n* = 40, 210 days old) were administered with the quantified fiber protein (HA titer of 11 log2) and inactivated EDS vaccine intramuscularly. Serum samples of laying hens were collected at 14, 21, and 28 dpi for HI test.

To investigate the correlation between HI titer and efficacy of subunit vaccine, first, laying hens in groups 1 to 5 (*n* = 40, 210 days old) were intramuscularly administered with purified fiber protein at the original volumes of 0.025, 0.05, 0.1, 0.2, and 0.4 mL (the injection volume for each group was adjusted to 0.4 mL by mixing with emulsified PBS), respectively. Forty unimmunized laying hens were used as the controls in group 6. Second, all the serum samples of hens in group 1 to 6 were collected for HI test at 28 dpi, and then all the laying hens were mixed; then reorganized into four groups (*n* = 30) based on the HI titer of 9, 8, 7, and 6 log2; and then orally challenged with HX strain (10^6.8^ EID_50_ per hen). Finally, all the challenged laying hens were monitored for 21 days to record egg production. The pale, thin-shelled, shell-less, or soft eggs were considered to be the abnormal eggs.

To investigate the correlation between the quantity of fiber protein and HI titer, laying hens in groups 1 to 3 (*n* = 40) were immunized with fiber protein at the HA titer of 11, 9, and 7 log2, respectively. The laying hens immunized with the inactivated EDS vaccine in group 4 were set as the positive control. The unimmunized laying hens in group 5 were set as the negative control. At 14, 21, 28, 60, 90, 120, 150, and 180 dpi, the serum samples of each group (*n* = 10, randomly selected) were collected for HI test.

### Statistical Analysis

Data were presented as mean ± SD. The differences in antibody titers and egg production ratio of laying hens between groups were determined using Student *t*-test. Differences were considered statistically significant when *p* < 0.05.

## Results

### Expression and HA Titer Test of Fiber Protein

**Figure 1:**
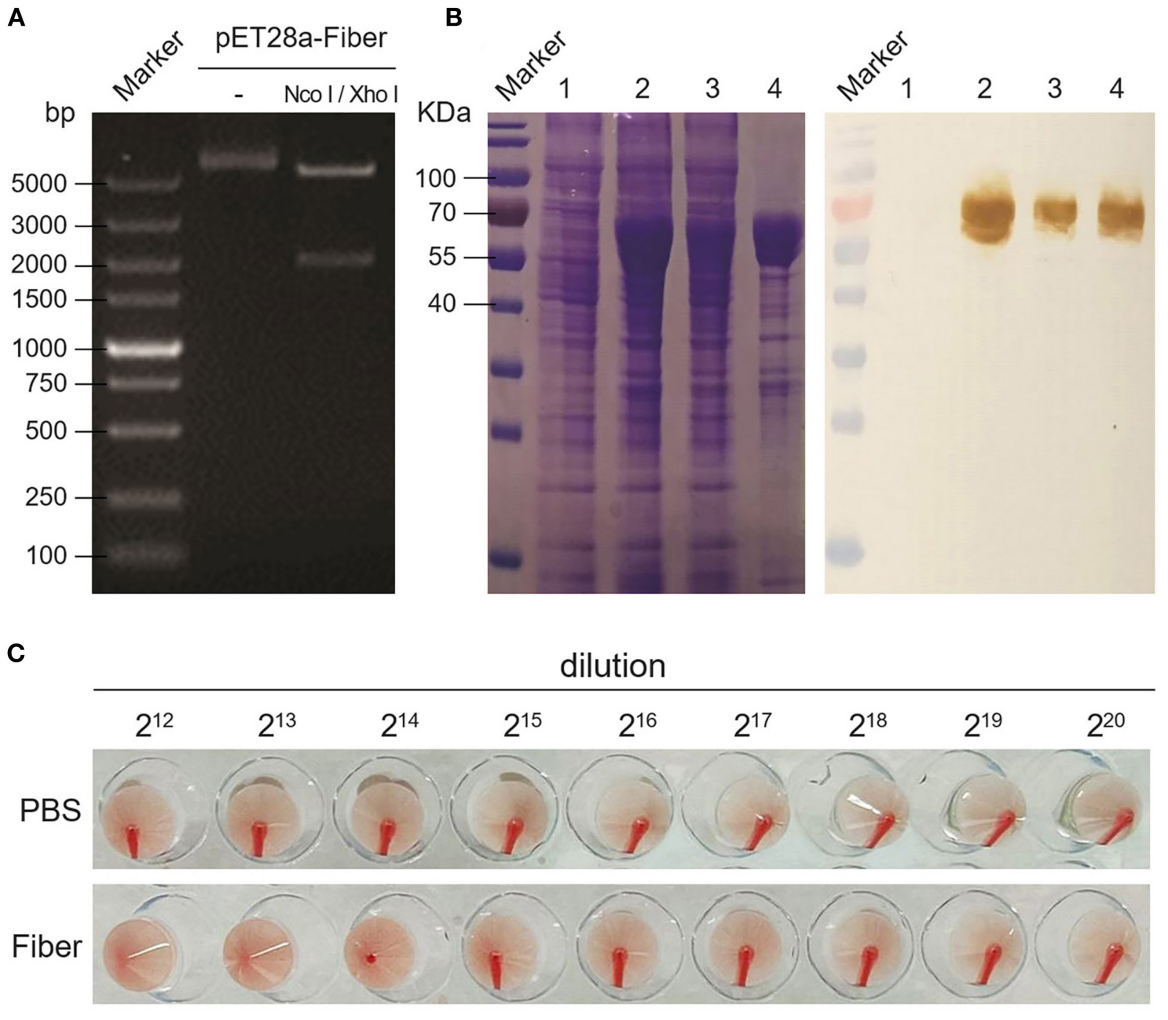
Expression and HA activity of DAdV-1 fiber protein. **(A)** Double restriction enzyme digestion of recombinant pET28a-fiber plasmid. **(B)** Expression and identification of fiber protein by SDS-PAGE and Western blot. Lane 1: bacteria without IPTG induction; lane 2: bacteria with IPTG induction; lane 3: supernatant of induced bacteria; lane 4: inclusion bodies of induced bacteria. Positive serum against DAdV-1 AV127 was used as primary antibody in Western blot. **(C)** HA activity test of fiber protein.

After plasmid construction (Figure 1A) and the expression in BL21 (DE3), the results of SDS-PAGE and Western blot showed a size of 68 kDa of fiber protein distributed in supernatant and inclusion body (Figure 1B), and the concentration is ~180 mg/L. After concentrating by the Ni-NTA Purification system, the results of HA assay showed the purified fiber protein (0.6 mg/mL) led to HA of the chicken erythrocyte (Figure 1C), with the highest HA titer of 13 log2 (Figure 1C).

### HI and Neutralization Activity of Serum From Immunized SPF Chickens

The results of HI and neutralization activity assays showed that both the fiber protein and inactivated EDS vaccine could induce the production of HI and neutralization antibodies (Table 1). At 14 dpi, the immunization of fiber protein induced an equal level of HI titer (9.7 ± 1.6 log2) in chickens compared with the inactivated vaccine (9.5 ± 1.5 log2), whereas at 21 and 28 dpi, the levels of HI titer induced by fiber protein were higher (11.1 ± 1.0 log2 and 12.5 ± 1.4 log2) than that of inactivated vaccine (10.1 ± 1.6 log2 and 11.5 ± 1.5 log2).

**Table 1:** HI and neutralizing antibodies titer tests of the serum samples collected from SPF chickens at 14, 21, and 28 days post immunization.

**Vaccine**	**HI mean titer (log2)**			**Serum neutralization (%)** ^a^		
	**14 d**	**21 d**	**28 d**	**14 d**	**21 d**	**28 d**
Fiber protein	9.7 ± 1.6	11.1 ± 1.0	12.5 ± 1.4	100	100	100
Inactivated EDS vaccine	9.5 ± 1.5	10.1 ± 1.6	11.5 ± 1.5	100	100	100
Control	0	0	0	0	0	0

a *Each number represents percent of eggs (out of six) in which the virus was neutralized*.

### Efficacy of Subunit Vaccine in Laying Hens

**Figure 2:**
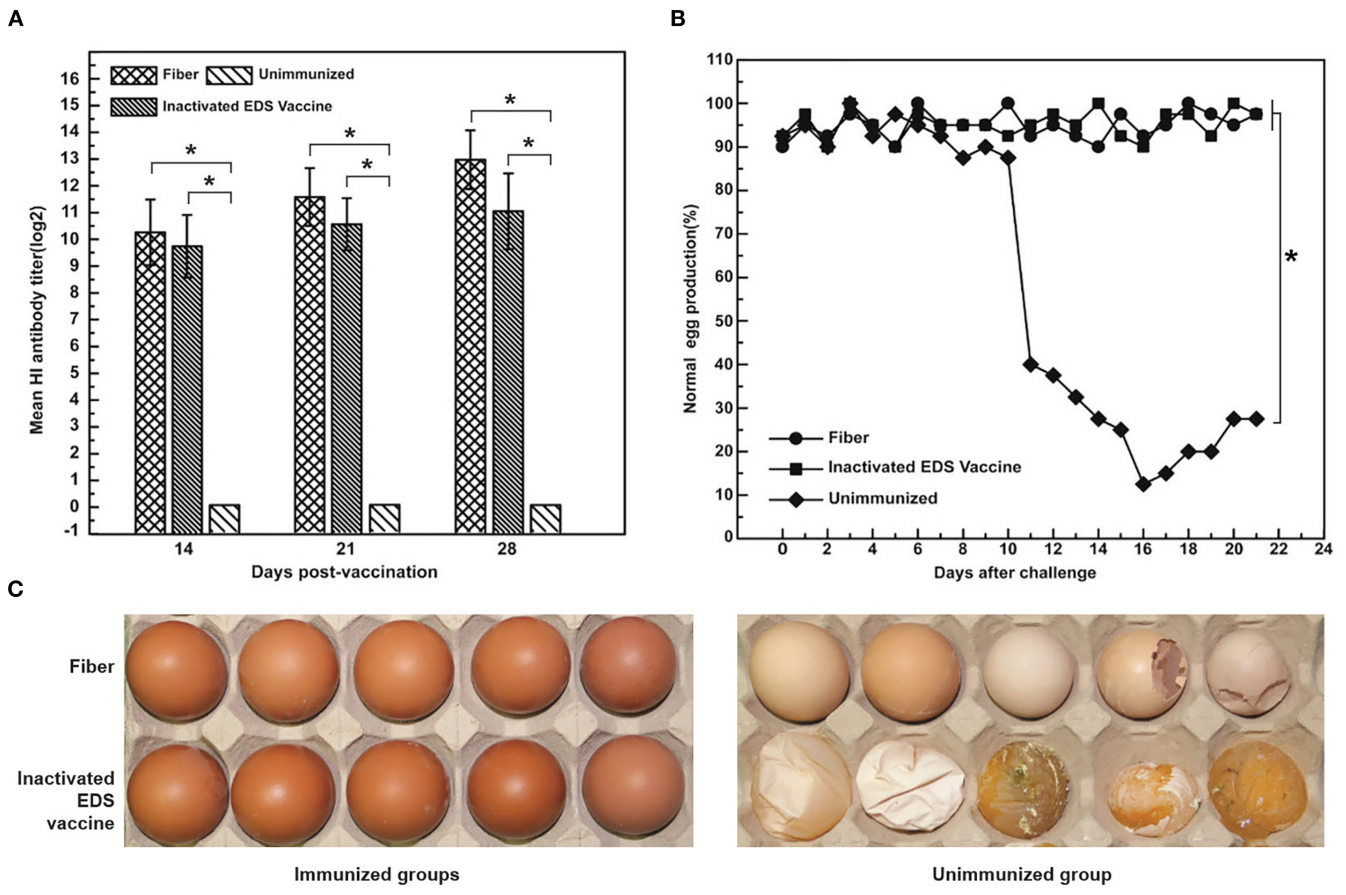
Assessment of vaccine efficiency in laying hens. **(A)** HI titer of the serum samples at 14, 21, and 28 dpi. **(B)** Egg production rate of the immunized laying hens after challenge with DAdV-1. **(C)** Comparison of normal eggs with pale, thin-shelled, shell-less, or soft eggs. The titers of antibodies are expressed as mean ± SD. Asterisk denotes a statistically significant difference (*p* < 0.05).

The results of subunit vaccine efficacy in laying hens showed that both the fiber protein and inactivated EDS vaccine could induce the equal levels of HI antibodies (Figure 2A). To test the efficacy of the fiber protein, laying hens were orally challenged with HX strain at 28 dpi, and then the egg production was recorded daily. At 11 days postchallenge (dpc), pale, thin-shelled, shell-less, or soft eggs were observed in the unimmunized group (Figure 2C); the egg production rate dropped to 12.5% at 16 days (Figure 2B). In contrast, there was no drop of egg production in the groups with fiber protein or inactivated EDS vaccine immunization (maintaining the egg production rate of 90–100% until 21 dpc) (Figure 2B). The results indicated that the fiber protein immunization reduces the impact of the drop in egg production caused by virus infection in laying hens significantly.

### Correlation Between HI Titer and Efficacy of Fiber Protein

**Figure 3:**
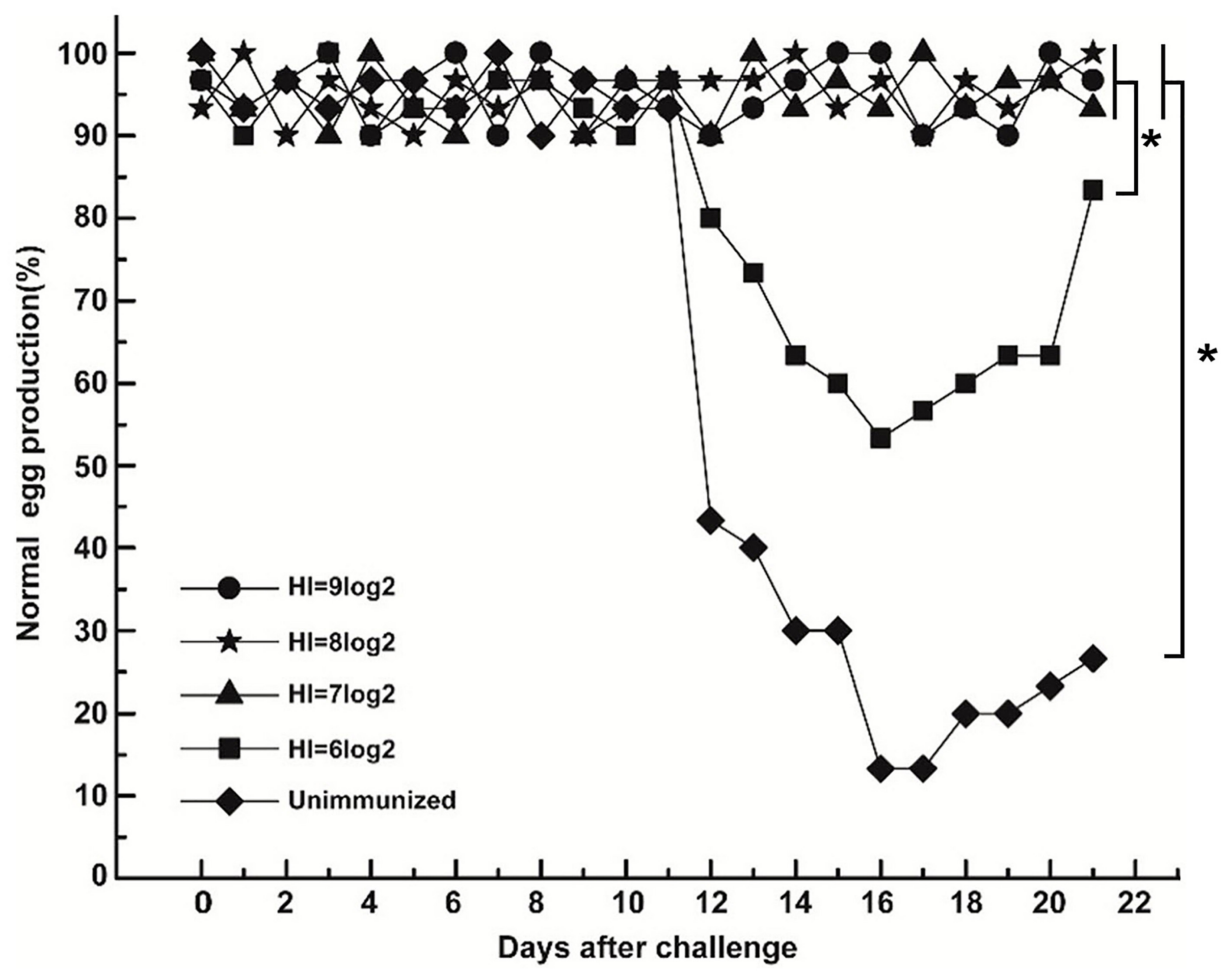
Correlation between HI titer and efficacy of the fiber protein. Based on HI titer, the immunized laying hens were regrouped to HI titer of 9, 8, 7, and 6 log2 and orally challenged with DAdV-1. Egg production rate was calculated daily. Asterisk denotes a statistically significant difference (*p* < 0.05).

After challenge, the egg production in the unimmunized group decreased from 12 dpc and dropped to 13% at 16 dpc (Figure 3). Meanwhile, egg production in the group with HI titer of 6 log2 decreased from 12 dpc and dropped to 53% at 16 dpc (Figure 3). In contrast, there was no drop of egg production in groups with HI titer of 9, 8, and 7 log2 until 21 dpc (Figure 3). The results indicate that the lowest HI titer for reducing egg production of laying hens against DAdV-1 infection is 7 log2.

### Antibody Persistence After Immunization of Fiber Protein With Different Antigen Contents

**Figure 4:**
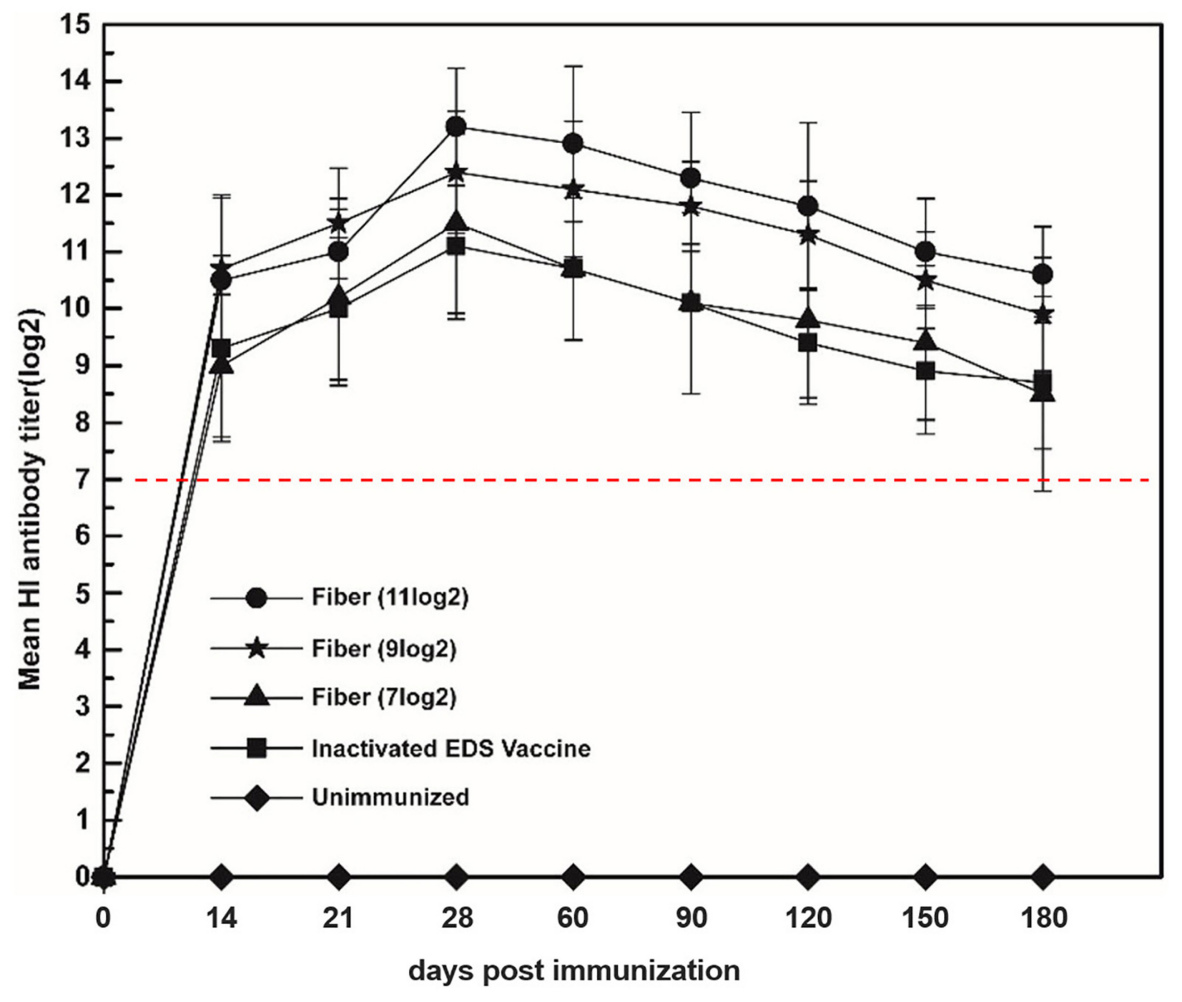
Correlation between fiber protein and HI titer induced by subunit vaccine. Laying hens were immunized with fiber protein at the HA titers of 11, 9, and 7 log2, respectively. The HI titers of the serum samples collected at 14, 21, 28, 60, 90, 120, 150, and 180 dpi were tested. The group immunized with the inactivated EDS vaccine was set as the positive control.

In the assay of the correlation between the quantity of fiber protein and HI titer, the results showed the HI titers induced by fiber protein and inactivated EDS vaccine ranged from 7 to 14 log2, which was higher or equal to the HI titer of 7 log2 that could give complete protection to laying hens against DAdV-1 infection (Figure 4). Moreover, the HI titer maintained in a high level until 180 dpi (Figure 4).

## Discussion

The knob domain of fiber protein contains most of the neutralizing epitopes of DAdV-1 and shows the ability to induce the production of HI and neutralizing antibodies (8), which make the fiber protein the most effective immune antigen and widely used in vaccine development (21–23). Previous studies showed that the truncated fiber protein produced as inclusion bodies in *E. coli* required dialysis and refolding, which was not suitable for further purification (21, 22). An improvement expression of truncated fiber protein was achieved by adding adjacent 60 amino acids of the shaft region to the knob domain and showed an HA titer of 1:512 (23), but a lack of further study in the clinical application in laying hens, and the correlation between fiber protein and HI titer was not clear. Here, the fiber gene of DAdV-1 HX strain showed a nucleotide identity of 99.5% (data not shown) to that of the standard DAdV-1 AV127 strain. Here, the HA titer (13 log2) of the fiber protein expressed in *E. coli* was higher than that in a previous report (23), which makes it suitable in the quantification of effective antigen.

Compared with inactivated EDS vaccine, the fiber protein could induce an equal level of HI and neutralizing antibodies, indicating good immunogenicity of the fiber protein. Serologically, the fiber protein was able to produce significantly higher protective antibody titer than that induced by whole viral DAdV-1 in the second week after vaccination (24). In laying hens, both the fiber protein and inactivated EDS vaccine provided complete protection against DAdV-1 infection. HA titer of fiber protein is the most direct manifestation of immune efficacy. The presence of invalid antigens in the proteins from different batches may cause differences in the effective immunogenic protein content; thus, besides the concentration, we thought it is more suitable to evaluate the clinical immunization effect when the HA titer was used as the immunization dose.

Previous studies showed that chicks that possessed maternal antibodies from infected hens conferred a passive immunity to challenge, based on the evidence of HI responses after challenge (25). Therefore, the level of HI antibody is regarded as an indicator of immune protection. Here, we found that the HI titer of 7 log2 is the lowest HI titer to reduce the egg production in laying hens against DAdV-1 infection. After challenge with DAdV-1, the lowest HA titer that could reduce the egg production in fiber protein–immunized laying hens is 7 log2, which induced the production of HI titer of 8 log2.

Meanwhile, the high level of HI antibodies even at 180 dpi makes the fiber protein suitable for long-term protection for laying hens against DAdV-1 infection. Previous studies showed that fiber protein could increase T-cell immunity via affecting T lymphocytes to control virus infection in SPF chickens (23), which deserved a further study in laying hens.

In summary, the fiber protein soluble expressed in *E. coli* showed the ability to induce the production of HI and neutralize antibodies. The lowest HI titer for complete protection of laying hens against DAdV-1 infection was 7 log2. Moreover, fiber protein with an HA titer of 7 log2 could induce an HI titer no <8 log2 in laying hens. Importantly, HI antibodies could be maintained at a high level up to 180 dpi. Overall, the fiber protein produced in *E. coli* is an effective subunit vaccine candidate in EDS ‘76 control in chickens.

## Data Availability Statement

The original contributions presented in the study are included in the article/Supplementary Material, further inquiries can be directed to the corresponding author/s.

## Ethics Statement

The animal study was reviewed and approved by the Research Ethics Committee of the National Research Center for Veterinary Medicine.

## Author Contributions

PZ and WL conceived and designed the research. LW, MW, HT, and PL conducted the experiments. PZ, LW, and WL analyzed the data. LW, PZ, WL, BH, and KT conceived the study, carried out additional analyses, and finalized the manuscript. All authors contributed to the article and approved the submitted version.

## Funding

This study was supported by the project of R&D and industrialization of genetically engineered vaccines for swine pseudorabies, swine ring and Mycoplasma hyopneumoniae (201200211200).

## Conflict of Interest

The authors declare that the research was conducted in the absence of any commercial or financial relationships that could be construed as a potential conflict of interest.

## Publisher's Note

All claims expressed in this article are solely those of the authors and do not necessarily represent those of their affiliated organizations, or those of the publisher, the editors and the reviewers. Any product that may be evaluated in this article, or claim that may be made by its manufacturer, is not guaranteed or endorsed by the publisher.

## Supplementary Material

Supplementary Figure 1 Schematic diagram of the expression plan for fiber protein. The amplified PCR fragment of the fiber gene was cloned into pET28a plasmid and then transformed into *E. coli*; the positive clone was cultured in LB medium by adding IPTG. The cell pellets resuspended were lysed by sonication and then centrifuged to remove the insoluble pellet. After that, the fiber protein was purified according to the instruction of Ni-NTA purification system.
