# Supplementary figures and images for: Fiber Protein Produced in Escherichia coli as a Subunit Vaccine Candidate Against Egg-Drop Syndrome 76
Source: Front Vet Sci. 2022 Feb 25;9:819217. doi: 10.3389/fvets.2022.819217 (PMC8913573; doi:10.3389/fvets.2022.819217)

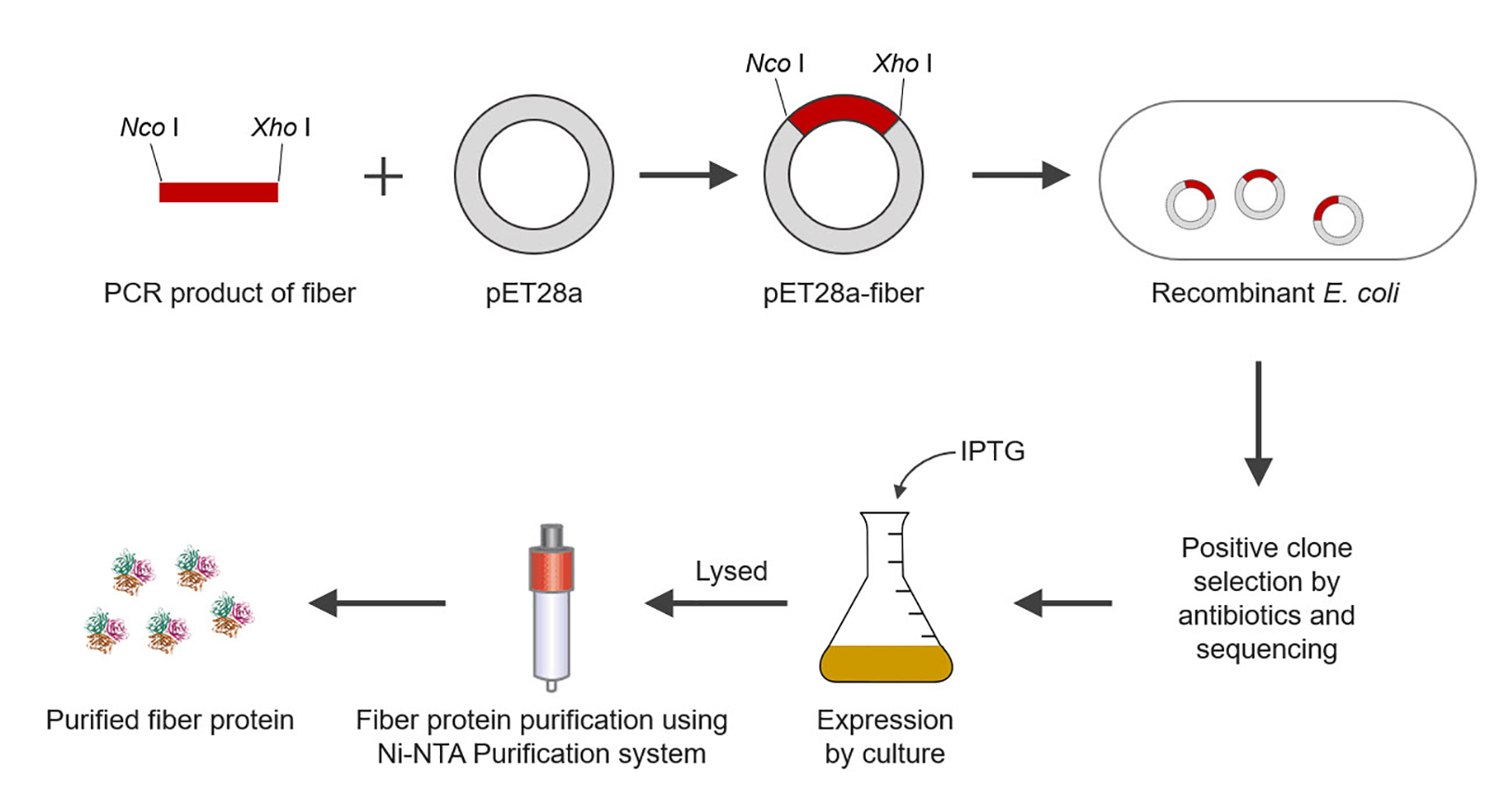

Supplement: Supplementary Figure 1 — Schematic diagram of the expression plan for fiber protein. The amplified PCR fragment of the fiber gene was cloned into pET28a plasmid and then transformed into E. coli; the positive clone was cultured in LB medium by adding IPTG. The cell pellets resuspended were lysed by sonication and then centrifuged to remove the insoluble pellet. After that, the fiber protein was purified according to the instruction of Ni-NTA purification system. [file Image_1.JPEG]
